# Fetal-derived PF4^+^ tissue-resident macrophages recruit neutrophils as a key mechanism underlying DENV-2-induced intrauterine growth restriction in mice

**DOI:** 10.1371/journal.ppat.1014524

**Published:** 2026-08-25

**Authors:** Hao Zhang, Wanchen Hao, Han Wang, Na Gao, Shiqi He, Ruiyang Li, Feiyang Xue, Dongying Fan, Yanhua Wu, Peigang Wang, Jing An, Ziyang Sheng

**Affiliations:** 1 Department of Microbiology, School of Basic Medical Sciences, Capital Medical University, Beijing, China; 2 Department of Blood Transfusion, Peking University Third Hospital, Beijing, China; 3 Laboratory for Clinical Medicine, Capital Medical University, Beijing, China; Pennsylvania State University College of Medicine: Penn State College of Medicine, UNITED STATES OF AMERICA

## Abstract

Extensive clinical cases and epidemiological analyses have indicated that symptomatic dengue virus (DENV) infection may lead to adverse outcomes during pregnancy; however, the precise mechanisms remain elusive. In our previous work, we demonstrated that DENV-2 infection induces intrauterine growth restriction by triggering neutrophil-mediated destruction of the placental vascular system in mice. However, the specific mechanisms driving neutrophil infiltration into the placenta remain unclear, especially given that the placenta constitutes a unique immune-privileged niche dedicated to maintaining maternal–fetal tolerance. In this study, we generated a time-resolved single-cell atlas of placentas from interferon-alpha/beta receptor-deficient mice infected with DENV-2. Integrated analysis of single-cell transcriptomes and single-nucleotide polymorphism sequencing revealed that infection primarily activated fetal-origin tissue-resident PF4 + macrophages. Activated fetal macrophages mediate preferential recruitment and the subsequent aberrant accumulation of neutrophils in the labyrinth zone through the secretion of CXCL2, which binds to neutrophil CXCR2. Notably, the recruited neutrophils further exhibited an enhanced self-recruitment phenotype. Blocking CXCL2-CXCR2 signaling effectively inhibited neutrophil recruitment, restored placental microvascular density, and alleviated fetal intrauterine growth restriction. This study revealed that the activation of placental tissue-resident macrophages is responsible for the aberrant neutrophil infiltration in the placenta following DENV-2 infection. Mechanistically, macrophages initiate the recruitment of self-amplifying neutrophils, and the CXCL2-CXCR2 signaling pathway plays a crucial role in this process. These results provide important information for the clinical development of therapeutic strategies against DENV-2-induced adverse pregnancy outcomes.

## Author summary

DENV infection during pregnancy can lead to poor fetal growth, but how the virus causes this problem is not well understood. The placenta, which supports the developing fetus, has a unique immune environment that normally prevents harmful inflammation. In this study, we used a mouse model to investigate what happens in the placenta after infection with DENV-2. We created a detailed map of individual cells in the placenta over time by combining two techniques: single-cell transcriptomics and SNP-based sequencing. This helps us distinguish whether immune cells originate from the mother or the fetus. We discovered that the virus primarily activates a group of fetal-origin, tissue-resident macrophages, which then release a chemical signal (called CXCL2) that attracts another type of immune cell—neutrophils—into the placenta. Once there, the neutrophils recruit even more neutrophils, creating a self-amplifying cycle of inflammation that damages the placental blood vessels. When the communication between the CXCL2 and its receptor on neutrophils were blocked, harmful neutrophil buildup could be prevented, followed by restored tiny blood vessels in the placenta, and improved fetal growth. Our study reveals a key role for fetal-origin macrophages in dengue-related pregnancy complications and points to a potential target for treatment.

## Introduction

Dengue virus (DENV) is a mosquito-borne pathogen that belongs to the genus Orthoflavivirus within the family Flaviviridae [1–3]. It is primarily transmitted by *Aedes aegypti* and *Aedes albopictus* mosquitoes [4]. In endemic tropical and subtropical regions, the geographic range and case numbers of DENV have continued to increase in recent years [5–7]. In recognition of its growing burden, the World Health Organization listed DENV as one of the top 10 threats to global health in 2019.

In addition to dengue fever, dengue hemorrhagic fever, and dengue shock syndrome, DENV infection during pregnancy has emerged as a significant clinical concern. Pregnant women with DENV infection are at increased risk of adverse outcomes, including miscarriage, preterm birth, and low birth weight. Epidemiological analysis has confirmed this association [8–11]. Pooled data have indicated that among pregnancies affected by DENV, the prevalence rates are 18.3% for preterm birth, 17.1% for low birth weight, 11.2% for small-for-gestational-age infants, and 3.3% for stillbirth, all of which are higher than those for uninfected pregnancies [12]. These findings, corroborated by a meta-analysis, have established a clear link between symptomatic maternal dengue infection and adverse outcomes [13]. Among these complications, intrauterine growth restriction (IUGR, clinically reflected as low birth weight or SGA) is the most frequently reported complication. IUGR is defined as the failure of a fetus to achieve its inherent growth potential. In practice, an infant with a birth weight below the 10th percentile for its gestational age is diagnosed with SGA, which is a major criterion for identifying IUGR [14,15]. This condition has serious short- and long-term health consequences for offspring, such as elevated childhood mortality and an increased propensity for adult metabolic disorders, including glucose intolerance [16,17]. Therefore, elucidating how DENV infection leads to pregnancy complications, particularly IUGR, is a pressing scientific need.

Given the pressing need to elucidate how DENV infection leads to pregnancy complications such as IUGR, research must focus on the placenta, as its dysfunction is a key determinant of such adverse outcomes [18,19]. The placenta forms a direct interface between the mother and fetus, playing critical roles in immune regulation while serving as a physical barrier [20]. In response to the semiallogeneic fetus, which functions as a “semiallograft” carrying paternal antigens, the maternal body develops a specialized microenvironment composed of two distinct compartments of different developmental origins: the maternal-derived decidua, containing heterogeneous cell types including decidual stromal cells, uterine natural killer cells, macrophages, dendritic cells, and T cells, and the fetal-derived chorion/placenta, compriseing various trophoblast subtypes such as cytotrophoblasts, syncytiotrophoblasts, and extravillous trophoblasts, as well as fetal endothelial cells [21,22]. This unique structure facilitates nutrient transport and immune protection while actively suppressing maternal immune rejection. Within this maternal-fetal immune microenvironment, placental neutrophils are key cellular components known to perform stage-specific functions, such as remodeling spiral arteries and initiating parturition [23,24]. Therefore, their recruitment and functional state must be tightly regulated.

The investigation into DENV-induced adverse pregnancy outcomes relies on animal models that replicate key human events such as fetal death and IUGR [25,26]. Previously, our laboratory established a model of DENV-induced IUGR using pregnant interferon-alpha/beta receptor-deficient mice [25]. In this model, the pathological characteristics included a marked reduction in placental microvessels accompanied by extensive neutrophil infiltration. Pharmacological inhibition of neutrophil degranulation was found to significantly restored placental microvascular density and alleviated fetal IUGR, indicating the central pathogenic role of neutrophils in DENV-induced placental vascular injury. However, the mechanisms governing neutrophil recruitment to placental tissue and the regulation of their function after the infection remain poorly understood. Therefore, further investigation into the mechanisms driving the pathological recruitment of neutrophils following DENV infection is crucial for developing future strategies to prevent or treat DENV-associated adverse pregnancy outcomes.

To further elucidate the mechanism of fetal IUGR induced by DENV-2 infection, we continued to explore the factors driving neutrophil chemotaxis to the placenta on the basis of this model. Our results suggest that fetal-derived tissue-resident macrophages play a key role in DENV-induced neutrophil recruitment and provide an important reference for the development of clinical therapeutic strategies against DENV infection-associated adverse pregnancy outcomes.

## Results

### DENV infection causes pronounced placental microvascular damage and IUGR at the late stage

**Fig 1:**
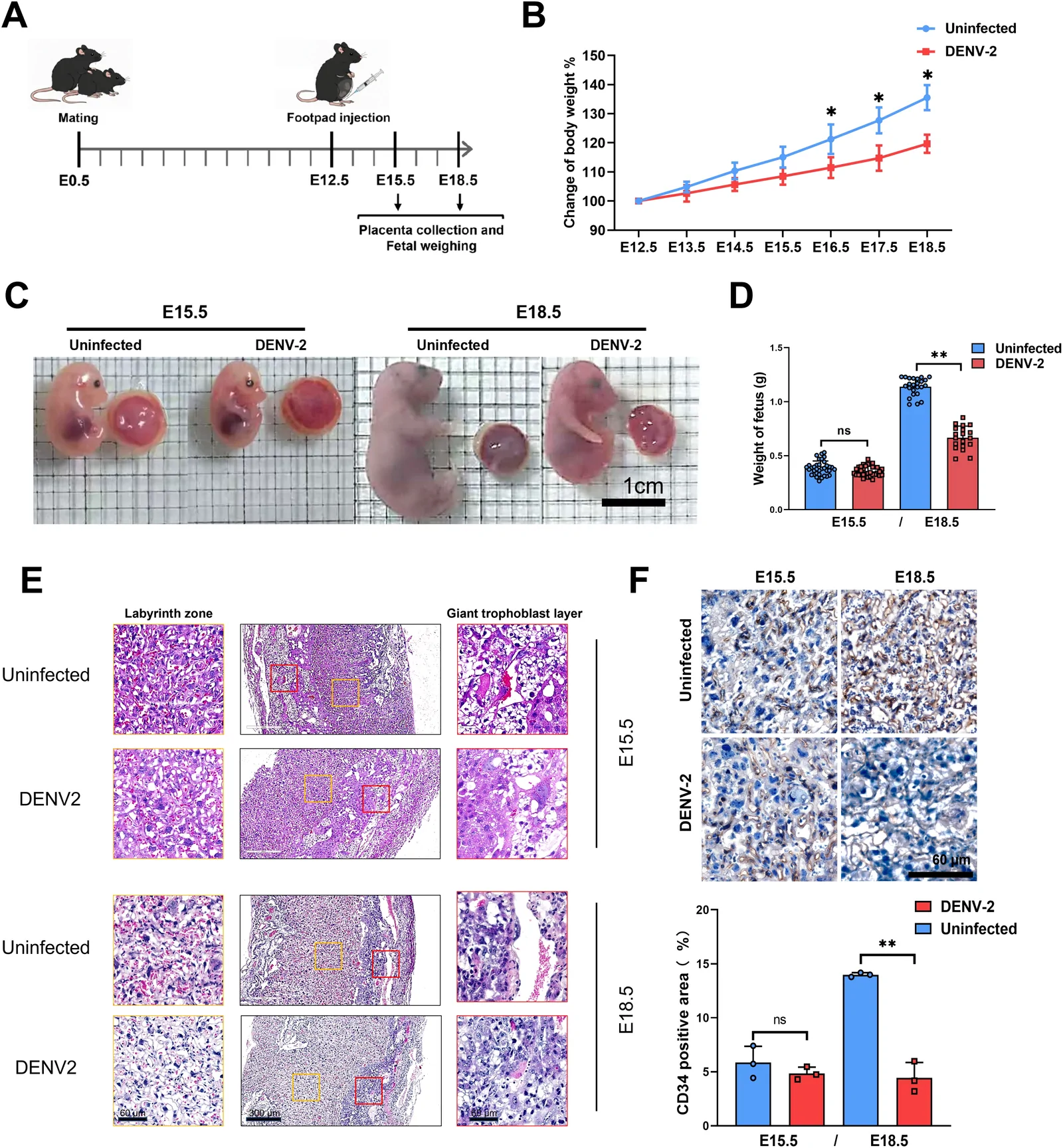
Neutrophil infiltration and microvascular reduction in the placental labyrinth occur in the late stage of DENV-2 infection. **(A)** Schematic diagram of the experimental procedure. Virgin *Ifnar1*^-/-^ female mice were mated with male mice. Gestational day (embryonic day 0.5, E0.5) was determined by vaginal plug observation. DENV-2 (10^5^ PFU) was administered by footpad injection at E12.5, fetuses and placentas harversted at E15.5 and E18.5 (3 and 6 days post-infection). **(B)** Gestational body weight changes in pregnant mice. Body weights before mating were set as 100%. Data was recorded until E18.5. n = 5. **(C)** Representative images of fetuses and placentas at E15.5 and E18.5. Scale bars indicate 1 cm. **(D)** Statistical analysis of fetal weight in E15.5 and E18.5. n = 5. **(E)** Typical images of placental structure and pathological changes in mice displayed by Hematoxylin and eosin (H&E) staining. Left (orange frame): 40 × view of placental labyrinth zone (scale bar: 60 µm). Middle (black frame): 8 × panoramic view (scale bar: 300 µm); the orange and red boxes indicate the locations of the 40 × fields shown in the left and right panels, respectively. Right (red frame): 40 × view of giant trophoblast layer (scale bar: 60 µm). **(F)** Representative images (left) and statistical analysis (right) of placental microvasculature in E15.5 and E18.5 using CD34 by immunohistochemical staining. Scale bar indicates 60 μm. In panel B,D and F, data was presented as the mean ± s.d. Repeated measures ANOVA was conducted for analysis of body weights **(B)**. Students’ t-test were used for statistical analysis of fetuses body weights **(D and F)**. *, p < 0.05; **, p < 0.01; ns, not significant.

We leveraged a previously established mouse model and explored the specific mechanisms through which DENV-2 infection leads to IUGR [25]. Pregnant A6 mice were infected with the DENV-2 strain Tr1751 via footpad injection at embryonic Day 12.5 (E12.5), while control mice received an equal volume of PBS (Fig 1A). Maternal body weight was recorded daily post infection. At E15.5 and E18.5, the mice were euthanized, and the placentas and fetuses were harvested for analysis.

The results showed that DENV-2 infection led to slower maternal weight gain. A significant difference in maternal weight between the infected and control groups became apparent from E16.5 onward (Fig 1B). At 3 days post-infection (dpi, E15.5), no significant changes in fetal weight or length were observed. However, by 6 dpi (E18.5), fetuses exhibited significant reductions in both weight and length, indicating the development of IUGR (Fig 1C and 1D).

Histological examination via H&E staining revealed a reduction in red blood cell perfusion within the placental labyrinth zone at E18.5 in the DENV-2-infected group, whereas no apparent change was detected at E15.5 (Fig 1E). Immunohistochemical staining for CD34 expression, a marker of microvessels, revealed a severe reduction in placental blood vessel density at E18.5 (Fig 1F). In contrast, placental microvascular density at E15.5 was largely unaffected by infection.

Collectively, these findings indicate that DENV infection-induced damage is more prominent during the later stage of infection.

### Single-cell sequencing reveals that placental mono/macrophage activation precedes neutrophil recruitment

**Fig 2:**
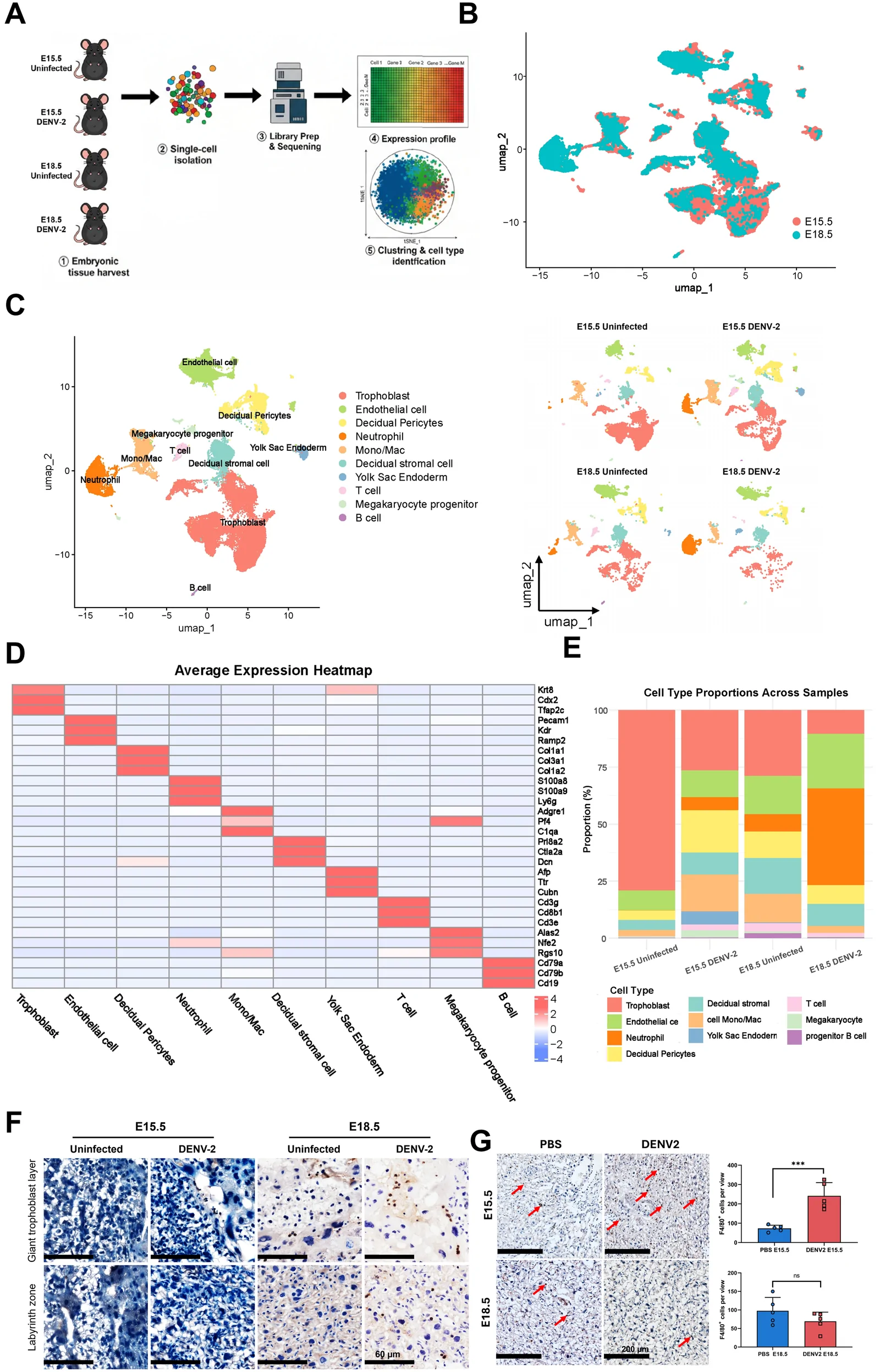
Single cell sequencing reveals earlier activation of placental mono-macrophages than neutrophils during DENV infection. **(A)** Experimental design for scRNA-seq analysis of placental cells from uninfected or DENV-2 infected mice at E15.5 and E18.5. Virgin *Ifnar1*^-/-^ female mice were mated with male mice. Gestational day (embryonic day 0.5, E0.5) was determined by vaginal plug observation. DENV-2 (10^5^ PFU) was administered by footpad injection at E12.5, fetuses and placentas harversted at E15.5 and E18.5 (3 and 6 days post-infection). Placentas were subjected for scRNA-seq analysis. **(B)** Integration and visualization of single-cell transcriptomes from E15.5 and E18.5 placentas using Harmony. **(C)** Uniform manifold approximation and projection (UMAP) plot showing 10 clusters cells in placentas (left). UMAP plots showing the distribution of placental cells from mice in different groups (right). **(D)** Heatmap showing the expression levels of representative cell type-specific marker genes for the corresponding cell types in the mouse placentas. **(E)** Relative changes in the cell ratios of placental cells across the 4 groups (Uninfected E15.5, DENV-2 infected E15.5, Uninfected E18.5 and DENV-2 infected E18.5). **(F)** Visualization of neutrophils in placental decidual and labyrinth zones by immunohistochemical staining using Ly6G. Scale bar indicates 60 μm. **(G)** Visualization (left) and statistical analysis (right) of placental macrophages by immunohistochemical staining using mice macrophages pan-marker F4/80. Scale bar indicates 200 μm. In panel G, data was presented as the mean and Students’ t-test was used for statistical analysis. *, p < 0.05; **, p < 0.01; ns, not significant.

To investigate the cause of placental microvascular damage at later stages of DENV-2 infection, we performed unsorted single-cell RNA sequencing (scRNA-seq) on placentas from infected and control mice at E15.5 and E18.5 (Fig 2A). After quality control, which involved removing cells with aberrant RNA counts or high mitochondrial gene content, the data were integrated using Harmony for batch correction and visualized via uniform manifold approximation and projection (UMAP). UMAP visualization revealed that cells from the two time points coembedded seamlessly within the shared clusters, indicating successful batch correction by Harmony. This enabled joint analysis of the combined dataset (Fig 2B). On the basis of canonical marker gene expression [27,28], we identified nine major cell types, namely, trophoblasts, endothelial cells, pericytes, neutrophils, monocytes/macrophages, stromal cells, yolk sac endoderm cells, T cells, B cells, and megakaryocyte progenitors (Fig 2C). A heatmap displays the expression of key marker genes for each cluster (Fig 2D). The relative proportions of each cell type at E15.5 and E18.5 were quantified (Fig 2E).

Comparisons with uninfected controls revealed an increase in the neutrophil proportion within infected placentas as early as E15.5 (Fig 2E). This finding was corroborated by Ly6G immunohistochemistry (IHC), which revealed detectable neutrophils in the maternal decidua of DENV-2-infected placentas at E15.5. By E18.5, neutrophils in control placentas were present and restricted to the decidua, likely reflecting a normal late-gestation pattern. In contrast, DENV-2 infection at E18.5 resulted in a substantial expansion of neutrophils, making them the most abundant cell type (Fig 2E). Notably, IHC revealed that the neutrophil distribution was no longer confined to the decidua but had expanded into the placental labyrinth zone (Fig 2F), indicating aberrant localization.

A more striking change was observed in the monocyte/macrophage population. At E15.5, their proportion increased substantially from 2.81% in controls to 16.20% in infected placentas, representing the most significant cellular increase at this time point (compared with 5.65% for neutrophils). By E18.5, however, the proportion of monocytes/macrophages decreased from 12.62% in the control placentas to 3.21% in the infected placentas (Fig 2E). This temporal pattern suggests early activation and potential subsequent depletion of placental monocytes/macrophages following infection.

IHC for the panmacrophage marker F4/80 validated these findings. The number of F4/80-positive cells was significantly greater in infected placentas than in control placentas at E15.5. By E18.5, compared with E15.5, IHC also revealed a decrease in placental macrophage numbers (Fig 2G). Together, these results indicate that at E15.5, monocyte/macrophage activation is significantly greater than neutrophil recruitment in the placenta in response to DENV-2 infection.

Given that monocytes/macrophages are key cells that interact with DENV in vivo, we analyzed viral RNA capture in our scRNA-seq data. DENV-2 nucleic acids were predominantly detected in monocyte/macrophage subpopulations (S1A Fig). The distribution and abundance of sequencing reads mapping to the DENV genome are shown in S1B Fig. No viral RNA was detected in the neutrophils, suggesting that DENV infection may not be the direct cause of neutrophil infiltration.

To further determine whether DENV directly infects placental cells, we performed immunostaining with anti-NS1 antibody and fluorescence in situ hybridization (FISH) on placental sections (S1C Fig). Consistent with our previous work, no viral antigen or nucleic acid was detected in the placenta [25]. These results collectively indicate that DENV-2 does not replicate in the placenta in our model; in other words, DENV-2 likely does not infect placental cells.

### CXCL2-CXCR2 axis is critical for neutrophil recruitment to the placenta following DENV-2 infection

**Fig 3:**
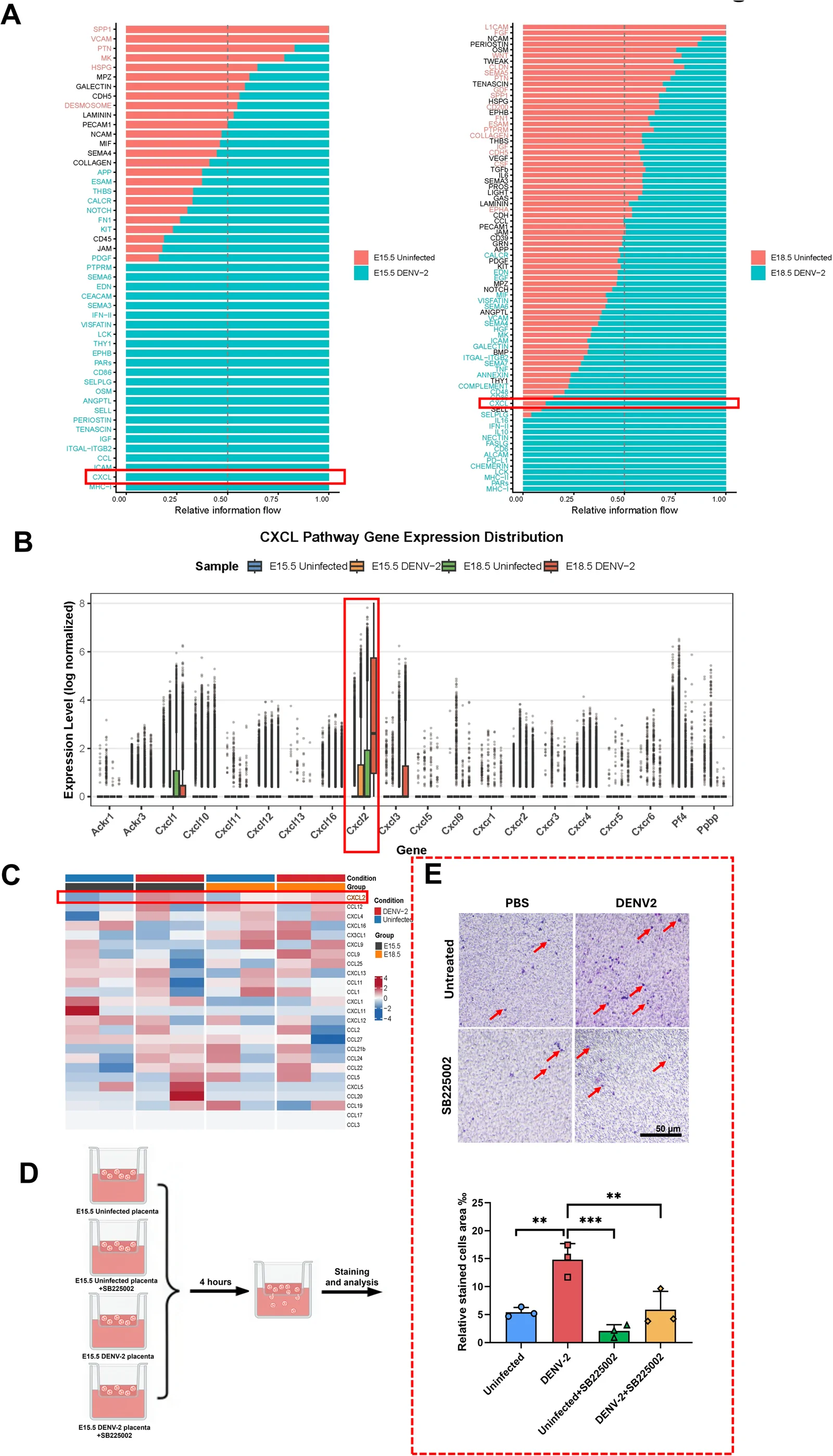
DENV-2 infection results in a significant increase of CXCL2 in the placenta. **(A)** Visualization of differences in the overall information flow for major signaling pathways, inferred by CellChat software from single-cell data of E15.5/E18.5 placentas. The information flow, representing the total communication probability within a network, is compared between uninfected (red) and DENV-2 infected (green) conditions. The red solid-line box outlines the CXCL signaling pathway. **(B)** Box plots showing the expression levels of individual molecules within the CXCL signaling pathway across all experimental groups. The red solid-line box outlines the cytokine gene *Cxcl2*. **(C)** Heatmap depicting the expression levels of chemokines in the placenta. The expression value for each chemokine is presented as a row-scaled Z-score. The red solid-line box outlines the cytokine CXCL2. **(D)** Schematic workflow of the experiment assessing the impact of CXCR2 inhibition on placenta induced neutrophil chemotaxis. Neutrophils isolated from mouse bone marrow were pretreated with either PBS (vehicle control) or SB225002 (a CXCR2 antagonist) and placed in the upper chamber. The lower chamber contained homogenized supernatant from either DENV-2-infected or uninfected placental tissue. **(E)** Representative images of migrating neutrophils on the transwell membrane and quantification of their numbers (n = 3 independent experiments). Scale bar: 50 µm. In panel E, data was presented as the mean and one-way ANOVA and Tukey HSD post-hoc test were used for statistical analysis. *, p < 0.05; **, p < 0.01; ns, not significant.

To characterize the aberrant immune activation within the placenta and investigate its potential connection with DENV-2 induced IUGR, we analyzed intercellular communication among the identified cell subsets at E15.5 and E18.5 using CellChat software. This analysis revealed a significant enhancement of the CXCL signaling pathway in infected placentas compared to controls at both E15.5 and E18.5 (Fig 3A). An analysis of the single-cell transcriptomics data focused on the CXCL signaling pathway revealed *Cxcl2* to be the most significantly differentially expressed gene, which displayed the most pronounced and sustained upregulation following infection (Fig 3B). Furthermore, a mouse chemokine array revealed that among the 25 chemokines analyzed, only CXCL2 had consistently increased expression at both E15.5 and E18.5 in infected placentas (Fig 3C). These findings suggest a potential key role of CXCL2 and the CXCL pathway in placental neutrophil recruitment.

To determine the role of the CXCL2-CXCR2 axis in placental neutrophil recruitment, we used a Transwell migration assay. Bone marrow-derived neutrophils were placed in the upper chamber, with supernatants from homogenized placental tissues (E15.5) serving as the chemoattractant source in the lower chamber. Neutrophil migration was significantly greater in supernatants derived from DENV-2-infected placentas than in those derived from control placentas, with the number of migrated cells quantified by crystal violet staining. To confirm the specific role of the CXCL2-CXCR2 pathway in this process, we pretreated neutrophils with SB225002, a pharmacological inhibitor of CXCR2. This intervention markedly attenuated the enhanced recruitment driven by infected placental supernatants (Fig 3D and 3E).

Collectively, these results demonstrate that the activation of the CXCL2-CXCR2 axis is a key mechanism driving neutrophil recruitment to the placenta following DENV-2 infection.

### Single-cell sequencing identifies Pf4 + macrophages as a major source of CXCL2

**Fig 4:**
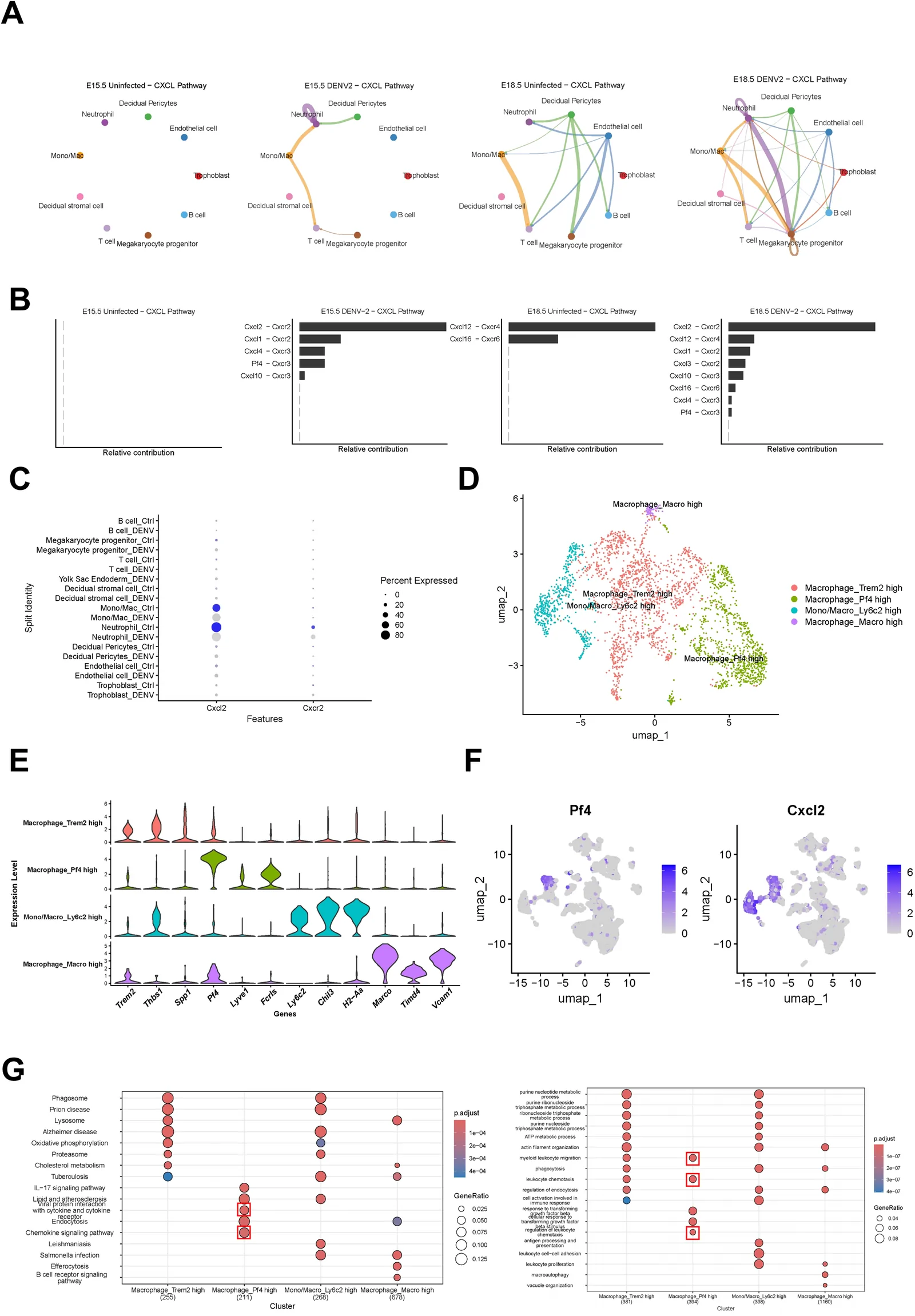
Single cell sequencing indicates that the Pf4 + macrophage subpopulation is the main source of CXCL2. **(A)** The inferred CXCL signaling network in E15.5 and E18.5 placenta. Circle sizes are proportional to the number of cells in each cell group (cluster). Edge widths represent the strength of communication probabilities. **(B)** Contribution plot showing the relative expression of ligand-receptor pairwise interactions within the CXCL signaling pathway across samples. **(C)** Dot plot showing the expression of Cxcl2 and Cxcr2 across different cell clusters. **(D)** UMAP visualization of Mono/macrophage from E15.5 mouse placenta. Cells are categorized into 4 distinct subtypes. **(E)** Violin plots showing the expression of highly expressed genes across Mono/macrophage subpopulations. **(F)** UMAP visualization of Cxcl2 and Pf4 gene expression across all E15.5 placental cells. **(G)** Dot plot of enriched KEGG pathways (left) and GO Biological Process (GO BP) terms (right) for each major Mono/macrophage subtype. The color of the dots represents the adjusted p-value, and the dot size corresponds to the gene ratio (proportion of expressed genes).

To investigate the cause of increased CXCL2 expression in placental tissue following infection, a detailed analysis of cellular communication was performed using CellChat. Neutrophils were identified as the primary recipient cells of CXCL signaling, while monocytes/macrophages were found to be a key source of these signals at both E15.5 and E18.5 (Fig 4A). Ligand–receptor pair analysis indicated that Cxcl2–Cxcr2 was consistently the dominant pair. Placental *Cxcl2* expression was primarily located in monocytes/macrophages and neutrophils, whereas *Cxcr2* expression was largely restricted to the neutrophil cluster (Fig 4B and 4C).

To more precisely define the role of distinct macrophage subpopulations in CXCL2 production, subclustering and dimensionality reduction were subsequently performed on the E15.5 monocyte/macrophage population, resulting in the resolution of four distinct subsets: Trem2 + , Pf4 + , Ly6c2 + , and Macro+ macrophages [29] (Fig 4D). Specific marker genes for each subset were visualized using violin plots (Fig 4E). A functional enrichment analysis (GO and KEGG) comparing these subsets revealed that the Pf4 + macrophage cluster was significantly enriched for terms related to the chemokine signaling pathway and virus–receptor interactions (KEGG), as well as myeloid cell chemotaxis and its regulation (GO Biological Processes) (Fig 4F). The enrichment profile strongly suggests that the Pf4 + macrophage subset is primarily responsible for secreting chemokines and orchestrating myeloid cell recruitment. In contrast, the Ly6c2-high subset was enriched for terms involving antigen presentation, leukocyte cell–cell adhesion, and leukocyte proliferation, suggesting its specialized role in initiating and maintaining adaptive immune responses. A feature plot revealed a high degree of coexpression between the key chemokine-encoding genes *Cxcl2* and *Pf4* in the macrophage population (Fig 4G).

Collectively, these data indicate that the Pf4 + macrophage subset is a major cellular source of placental CXCL2 during DENV-2 infection.

### SNP sequencing identifies Pf4 + tissue-resident macrophages as fetally derived

**Fig 5:**
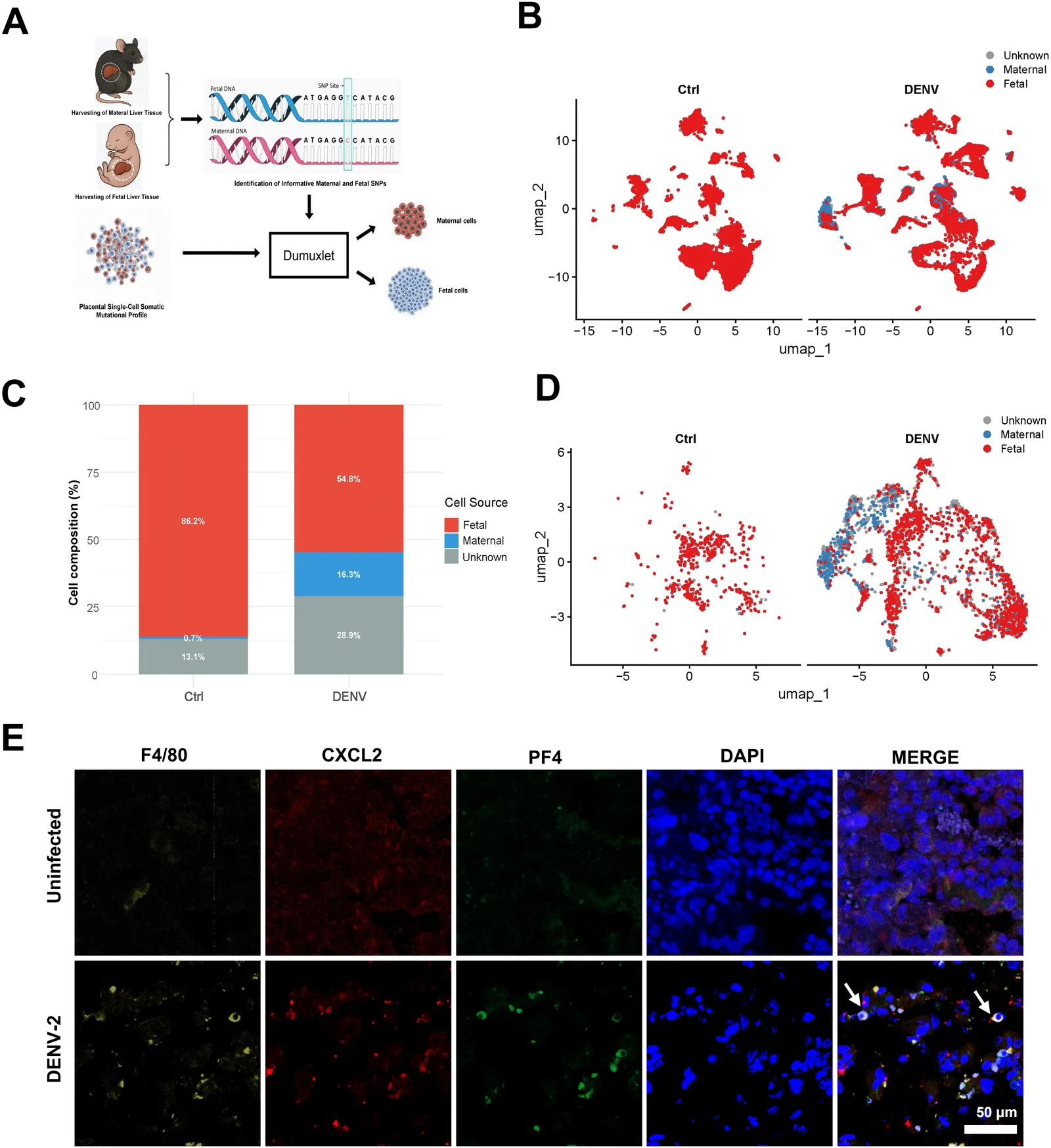
SNP sequencing confirms that PF4 + tissue resident macrophages are of fetal origin. **(A)** Schematic of the experimental design for demultiplexing placental cell origins using combined single-cell RNA and SNP sequencing. Maternal and fetal SNP profiles were obtained from liver tissues. The cellular genetic origin (maternal vs. fetal) of individual placental cells was then inferred based on the presence of unique, parent-specific SNP alleles. **(B)** UMAP visualization of E15.5 placentas shows qualitatively concordant cellular distributions after SNP-based demultiplexing between uninfected and DENV-2 infected conditions. This indicates that the overall spatial organization and the mixing pattern of maternal versus fetal-derived cells are largely preserved post-infection. **(C)** Stacked bar plot showing the proportion of cells identified as maternal or fetal in origin in uninfected and DENV-2 infected placentas. **(D)** UMAP visualization of macrophage subpopulations in E15.5 placenta, colored by their SNP-inferred cellular origin (maternal vs. fetal), across both uninfected and DENV-2 infected conditions. **(E)** Multiplex immunohistochemistry of E15.5 placental tissues from uninfected and DENV-2 infected mice. Sections were stained for F4/80 (650 dye, yellow), PF4 (520 dye, green), and CXCL2 (570 dye, red). Representative immunofluorescence images show CXCL2 staining within PF4^+^ macrophages (indicated by white arrows). Scale bar indicates 50 μm.

The placenta harbors two distinct mononuclear phagocyte systems of maternal and fetal origins. Studies suggest these populations play unique roles in responding to pathogenic infection, yet methods to definitively distinguish them are lacking. In this study, SNP sequencing was employed to determine the cellular origin within E15.5 placentas (Fig 5A). SNP anlysis revealed that placentas from uninfected mice were composed almost exclusively of fetally derived cells. Following DENV-2 infection, a notable increase in maternally derived cells was observed, although fetal cells remained the majority (Fig 5B and 5C). SNP analysis of the placental Mono/Mac cluster indicated that among its subsets, only the Ly6c2^+^ population was maternally derived [29]. In contrast, the Trem2^+^, Pf4^+^, and Macro+ subsets were all identified as fetally derived macrophages (Fig 5D).

Given that single-cell analysis identified Pf4^+^ macrophages as the principal subset that regulates myeloid cell chemotaxis in the placenta, multiplex immunohistochemistry was performed to investigate the presence of PF4^+^F4/80^+^CXCL2^+^ macrophages. The results confirmed the presence of macrophages coexpressing PF4, F4/80, and CXCL2 within the labyrinth zone of DENV-2-infected placentas at E15.5 (Fig 5E). Together, these analyses reveal that Pf4 + macrophages are characterized as both fetally derived and spatially localized within the placental labyrinth. These findings explain the cause of aberrant neutrophil infiltration into the labyrinth zone following DENV-2 infection.

In addition, analysis of our single-cell data via CellChat indicated that placental neutrophils transmit self-recruiting signals post infection (Fig 4A), which aligns with observations that human placental neutrophils can undergo self-recruitment. Quantification and comparison of the contributions of monocytes/macrophages and neutrophils to neutrophil recruitment signals revealed that neutrophil-derived signaling became the predominant source of CXCL signaling by E18.5 (S2A Fig). Immunohistochemistry confirmed the presence of CXCL2 + neutrophils in DENV-2-infected placentas (S2B Fig). Flow cytometry demonstrated a significant increase in the number of CXCL2 + Ly6G+ cells in infected placentas (S2C and S2D Fig). Finally, by isolating neutrophils from uninfected and DENV-2‑infected placental tissues, we assessed their ability to recruit naïve bone marrow‑derived neutrophils. The results showed that neutrophils from infected placentas exhibited a significantly enhanced recruitment capacity (S2E Fig). These results suggest that neutrophils in DENV-2-infected placentas can also recruit additional neutrophils. Collectively, these data show that macrophage-driven recruitment and subsequent self-recruitment of neutrophils create an amplifying cascade and that CXCL2 is central to maintaining this process.

### The placentas of DENV-infected women harbour PF4 ⁺ fetal macrophages and exhibit IL-8/CXCR2 expression

**Fig 6:**
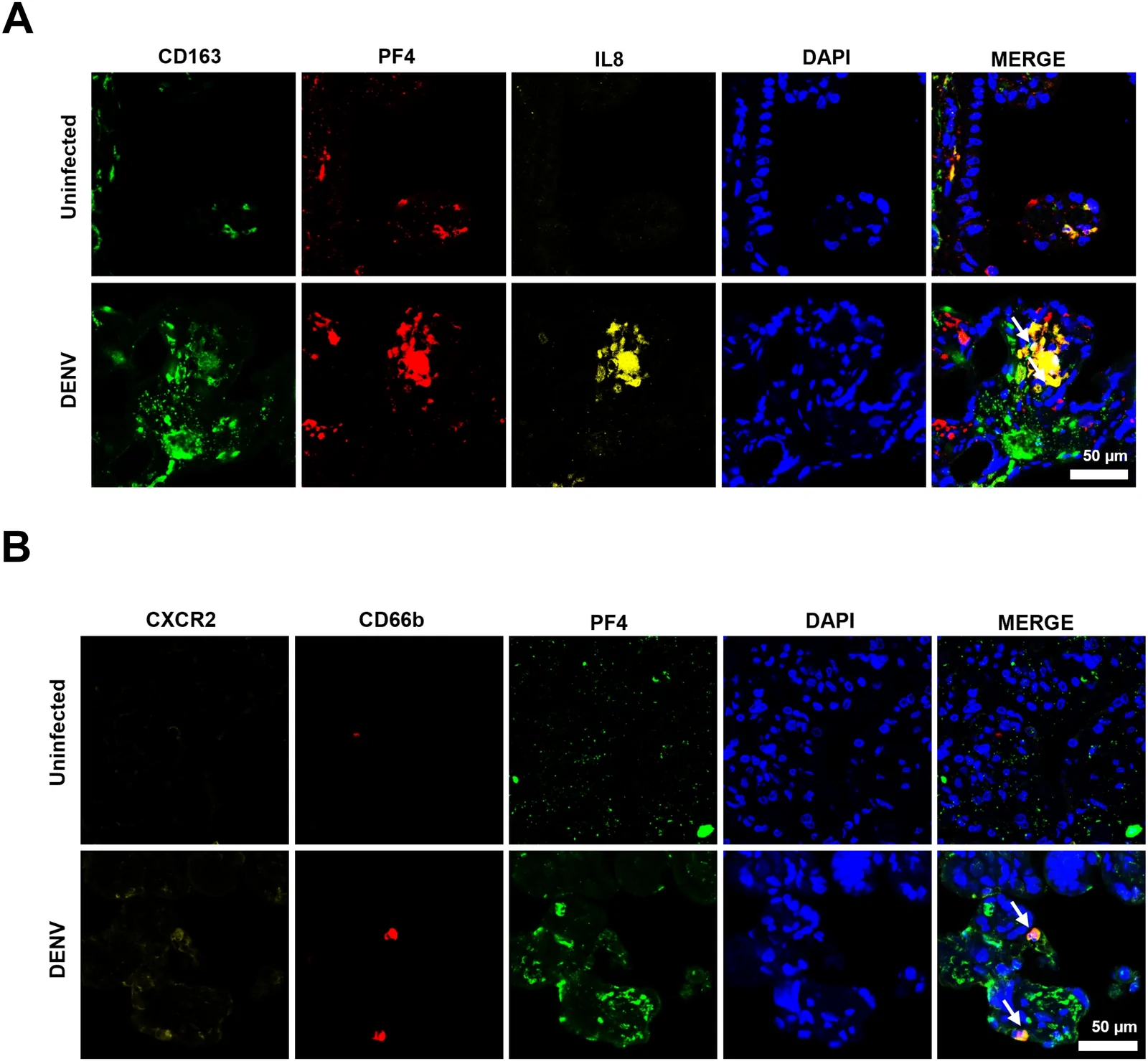
Multiplex immunohistochemistry (mIHC) staining for detecting expressions of IL8 in PF4 ⁺ cells and CXCR2 in CD66b⁺ cells in human placental samples. **(A)** Multiplex immunohistochemistry staining of human placental tissues from healthy and DENV infected woman. Sections were stained for IL8 (650 dye, yellow), CD163 (520 dye, green), and PF4 (570 dye, red). Representative immunofluorescence images show IL8 staining within PF4^+^ macrophages (indicated by white arrows). Scale bar indicates 50 μm. **(B)** Multiplex immunohistochemistry of human placental tissues from healthy and DENV infected woman. Sections were stained for CXCR2 (650 dye, yellow), PF4 (520 dye, green), and CD66b (570 dye, red). Representative immunofluorescence images show CXCR2 staining within CD66b^+^ neutrophils (indicated by white arrows). Scale bar indicates 50 μm.

Placental tissue sections from DENV‑infected pregnant women and healthy controls were examined by multiplex immunohistochemistry staining. PF4 ⁺ CD163 ⁺ cells were detected in both uninfected and DENV‑infected human placentas, indicating that PF4 ⁺ fetal macrophages reside in the human placenta (Fig 6A). We then examined IL‑8 (the functional counterpart of mouse CXCL2) expression in PF4 ⁺ macrophages and found that IL‑8 ⁺ PF4 ⁺ cells were present only in DENV‑infected placentas, but not in uninfected controls. Using CD66b to label neutrophils, we also assessed CXCR2 expression on these cells and observed that CXCR2 ⁺ neutrophils were found exclusively in DENV‑infected placentas (Fig 6B). Together, these results suggest that DENV infection during pregnancy may induce IL‑8 production by PF4 ⁺ fetal macrophages and promote CXCR2‑mediated neutrophil recruitment, consistent with the findings from our mouse model.

### Inhibition of CXCL2-CXCR2 signaling reduces placental neutrophil infiltration and alleviates IUGR

**Fig 7:**
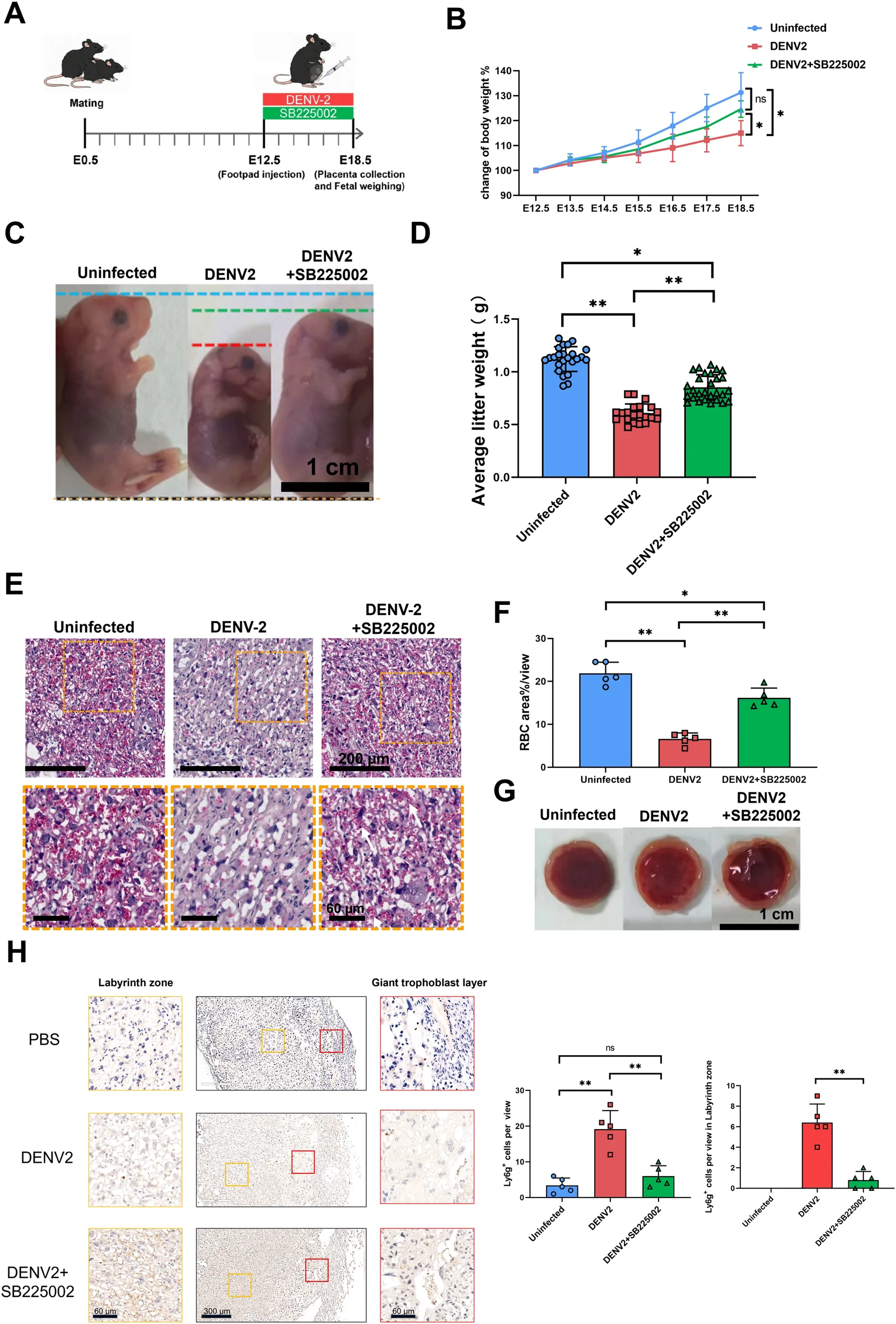
Inhibiting CXCL2 signaling reduces neutrophil infiltration and alleviates intrauterine growth restriction in mice. **(A)** Schematic of experimental design. Virgin female *Ifnar1*^-/-^ mice were mated with sexually mature fertile male mice. Gestational day was defined as embryonic day 0.5 (E0.5) by vaginal plug observation. DENV-2 infection was performed at E12.5, followed by daily intraperitoneal injection of the CXCR2 inhibitor SB225002 (5mg/kg/day) until E18.5. **(B)** Maternal body weight changes during pregnancy. The weights of pre-infection mice at E12.5 were set as 100%. Data was recorded until the experimental endpoint (E18.5). n = 5. **(C)** Representative images of fetuses at E18.5. Scale bar indicates 1 cm. **(D)** Statistical analysis of fetal weight at E18.5. n = 5. **(E)** Placental labyrinth zone displayed by Hematoxylin and eosin (H&E) staining. White arrows indicate areas of restored placental blood supply. Scale bar indicates 200 or 60 μm. **(F)** Statistical analysis of red blood cell area (%) by image J software. n = 5. **(G)** Representative images of placentas at E18.5. Scale bar indicates 1 cm. **(H)** Visualization of neutrophils in placentas by immunohistochemical staining using Ly6G. Left (orange frame): 40 × view of placental labyrinth zone (scale bar: 60 µm). Middle (black frame): 8 × panoramic view (scale bar: 300 µm); the orange and red boxes indicate the locations of the 40 × fields shown in the left and right panels, respectively. Right (red frame): 40 × view of giant trophoblast layer (scale bar: 60 µm). Statistical analysis of the number of neutrophils by Image j software. n = 5. In panel B,D,F and H, data was presented as the mean ± s.d. Repeated measures ANOVA was conducted for analysis of body weights **(B)**. One-way ANOVA and Tukey HSD post-hoc test were used for statistical analysis in panel D, F and H. *, p < 0.05; **, p < 0.01; ns, not significant.

To validate the role of CXCL2-CXCR2 in recruiting neutrophils to the placenta and inducing IUGR following DENV-2 infection, pregnant A6 mice were treated with the classic CXCR2 inhibitor SB225002. The mice received daily intraperitoneal injections of the inhibitor at 5 mg/kg body weight, as outlined in the schematic (Fig 7A).

Maternal body weight was monitored throughout pregnancy. The inhibition of CXCL2 effectively mitigated DENV-induced maternal weight loss, with a statistically significant improvement observed from E15.5 onward compared with that in the infected, untreated group (Fig 7B). At E18.5, compared with those from the DENV-2 infection group, fetuses from the SB225002-treated group showed significant recovery in terms of both length and weight. Statistical analysis confirmed that fetal weight was significantly greater in the SB225002-treated group than in the DENV-2-infected group (Fig 7C and 7D).

Histological examination of the placentas revealed that CXCL2 inhibition restored red blood cell perfusion in pregnancies affected by DENV-2, effectively increasing the perfused area within the placental labyrinth (Fig 7E and 7F). Gross examination revealed that the placentas from SB225002-treated mice were markedly more reddish in color than the pale placentas from the DENV-2 infection group, closely matching the appearance of those from the uninfected controls (Fig 7G). Immunohistochemical staining for Ly6G was performed to assess neutrophil infiltration in Giant trophoblast layer and Labyrinth zone. A marked reduction in placental neutrophil infiltration was observed in mice receiving the CXCR2 inhibitor compared with that in the untreated infection group (Fig 7H). Together, these results demonstrate that *in vivo* blockade of CXCL2 is sufficient to reduce the aberrant placental neutrophil infiltration caused by DENV-2 infection and effectively alleviate the associated IUGR.

## Discussion

DENV infection during pregnancy is highly concerning because of its association with adverse outcomes such as miscarriage, stillbirth, and IUGR [8,12,13]. However, the underlying mechanisms remain poorly understood. Owing to similarities in chorionic-placental structure and physiology, mouse models are widely used to study the pathogenesis of human pregnancy disorders [30,31]. In our prior work, severe neutrophil infiltration and placental microvascular loss were observed in DENV-2-infected mice; however, the mechanism driving this localized neutrophil recruitment was not elucidated [25]. The results of this study revealed that in infected mice, the marked increase in placental neutrophils coincided with a reduction in microvascular density, both of which occurred at relatively late stages. More critically, after DENV-2 infection, recruited neutrophils accumulated in the labyrinth zone, which is a highly vascularized region critical for maternal–fetal exchange. This accumulation was not observed under normal conditions.

Importantly, although single-cell transcriptomics captured viral reads covering nearly the full length of the DENV genome in placental macrophages, we did not detect viral antigen or RNA in placental sections by staining with anti-NS1 antibody or FISH, being consistent with our previous work. This suggests that macrophages likely phagocytose DENV-2 particles without supporting active viral replication, resulting in viral nucleic acid and antigen levels below the detection thresholds of RNA FISH and antigen staining. Collectively, these findings indicate that there is no DENV-2 transplacental transmission in our model, and the viral signals captured by scRNA-seq more likely to reflect phagocytic events rather than authentic viral replication.

Collectively, these findings support the conclusion that neutrophils act as critical mediators in the pathogenesis of DENV-2-induced adverse pregnancy outcomes. This pathogenic role may extend to clinical disease, as elevated peripheral neutrophil counts in dengue patients are similarly associated with disease severity, where neutrophil-derived mediators such as IL-8 and TNF-α, along with transendothelial migration and NETosis [32,33]. The latter is a form of neutrophil cell death, and our recent studies have demonstrated its ability to induce microvascular damage, thereby providing a mechanistic explanation for the vascular pathological changes observed in DENV infection [34]. Notably, the robust neutrophil recruitment observed in our DENV-2 model constitutes a distinct feature compared to ZIKV infection in placenta (S3 Fig). This response differentiates DENV infection from other vertically transmissible pathogens known to cause placental damage, including ZIKV, HIV-1, HCMV, and HCV, none of which are typically associated with substantial placental neutrophil infiltration [35–37]. Taken together, these findings suggest that neutrophil-mediated vascular disruption represents a pathological mechanism that is particularly prominent in, if not unique to, DENV-induced pregnancy complications.

Neutrophil migration along chemokine gradients is crucial for infiltration into sites of infection and chemokine dysregulation is a recognized driver of pathological neutrophil infiltration across diverse diseases, although the specific chemokine involved varies by context [38,39]. For example, CXCL1 mediates CXCR2 + neutrophil migration in models of multiple sclerosis, whereas CCL5 is required for hippocampal neutrophil recruitment in depression [40,41]. Analysis of placental inflammatory factors in our model revealed that the expression of CXCL2, the sole chemokine, was consistently and significantly upregulated. CXCL2 is a ligand for CXCR2, and their interaction is key for neutrophil activation and recruitment. Blockade of the CXCL2-CXCR2 axis with a CXCR2 inhibitor effectively reduced placental neutrophil infiltration, particularly in the labyrinth zone; diminished vascular damage; improved the placental blood supply; and alleviated fetal IUGR. These *in vivo* results confirm the pivotal role of CXCL2 in DENV-2-mediated adverse pregnancy outcomes. Notably, in human clinical cases, CXCL8 (mCXCL2 is the functional homolog of hCXCL8) has been reported to be specifically and highly expressed following DENV-2 infection and is recognized as a significant pathogenic factor [42,43]. Consistently, our immunostaining of human placental tissues from DENV-infected pregnancies revealed the presence of cells with high expression of CXCL8 and its receptor CXCR2, whereas no such staining was observed in placentas from healthy pregnant women. In the present study, we observed that CXCL2 expression was persistently elevated in the placentas of DENV-2-infected mice. These findings closely align with the clinical characteristics of DENV-2 infection in humans, effectively linking the mechanistic insights from our mouse model to human disease pathology. Besides, our single-cell transcriptomic analysis revealed a trend toward upregulation of NLRP3 and IL1β in the DENV-infected placenta, suggesting a potential link between inflammasome activation and the robust neutrophil infiltration observed in our model. However, the functional role of this pathway in mediating DENV-induced placental inflammation remains to be fully elucidated, and detailed mechanistic studies including validation at the protein level, are ongoing and will be reported in future work (S3 Fig). Taken together, these findings support the conclusion that aberrantly increased CXCL2 expression is a key driver of pathological neutrophil infiltration into the placenta.

We next sought to identify the cellular source of CXCL2 after DENV-2 infection. In our model, neutrophils were scarcely detected in the placenta at E15.5 but were present at E18.5, suggesting that at the earlier time point, neutrophils are not a resident placental population and that their recruitment is likely mediated by other cells. This inference was further supported by the absence of detectable viral RNA in neutrophils, indicating that their infiltration is unlikely to be a direct response to viral infection but rather results from the activation of other cell types. Our scRNA-seq data revealed a significant expansion of monocyte/macrophage populations at E15.5 following DENV-2 infection, which coincided with increased CXCL2 signaling, indicating that these cells are involved in initial neutrophil recruitment. The placenta contains two independent monocyte/macrophage systems of maternal (PAMMs) and fetal (Hofbauer cells) origin, which play distinct roles in immune defense at the maternal–fetal interface [44]. While they are crucial for protection, these cells can also be hijacked by pathogens such as ZIKV and HCMV for replication, disrupting immune tolerance and causing inflammation [45,46].

To date, discriminating maternal from fetal macrophages in the placenta remains challenging. Here, SNP sequencing coupled with scRNA-seq analysis was employed to determine cellular origin on the basis of genotype-specific single nucleotide polymorphisms [47]. This approach revealed that the high-CXCL2-expressing population consisted predominantly of fetal-derived, PF4^+^ fetal macrophages localized in the labyrinth. These findings explain why neutrophils are recruited specifically to this region, leading to localized microvascular damage. Although PF4^+^ fetal macrophages have been previously described in the placenta [28], this study is the first to implicate this subset in pathogenesis. Importantly, in human placental tissues, we also detected PF4^+^CD163^+^ macrophages, suggesting the presence of a similar cell population in humans and potential clinical relevance.

Given that DENV naturally infects only humans and non-human primates, mice with deficiencies in type I or combined type I and II interferon receptors have been widely adopted in studies of DENV pathogenesis and vaccine evaluation because of ethical and animal welfare considerations [48,49]. Currently, no superior model exists to replace them. Nevertheless, we acknowledge that impaired interferon responses may still amplify the pathological effects of DENV-2 on the placenta and fetus, which constitutes a key limitation of this study. Therefore, while the mechanistic findings presented here are compelling within this model system, they warrant further validation in clinical cohorts to confirm their relevance to DENV-2 infection in humans during pregnancy.

In conclusion, through integrated single-cell sequencing, SNP sequencing, and functional validation in a mouse model, this study elucidated a key mechanism for DENV-2-induced adverse pregnancy outcomes. We found that in DENV-2 infected placenta, fetal-derived, placental-resident Pf4 + macrophages to secrete CXCL2, which drives aberrant neutrophil recruitment into the placental labyrinth. This leads to cumulative neutrophil-mediated microvascular destruction and subsequent IUGR. Our findings reveal a previously unrecognized form of maternal–fetal immune crosstalk mediated by fetal-derived placental PF4 ⁺ macrophages, providing new insight into immune regulation at the maternal–fetal interface during viral infection. This mechanism offers a direct explanation for DENV-2-associated intrauterine growth restriction, lays a translational foundation for potential therapeutic strategies for DENV-induced pregnancy, and provides a broader perspective for studying how other viruses may impact gestation.

## Materials and methods

### Ethics statement

All the animal experimental protocols used in this study were approved by the Experimental Animal Welfare and Ethics Committee of Capital Medical University, Beijing, China.

The human placental tissue sections used in this study were obtained from our previously published study and were approved by the Medical Ethics Committee of Guangzhou Eighth People’s Hospital (approval No. 201508020263) [25]. DENV infection was confirmed by the following criteria: positive detection of serum DENV NS1 antigen and DENV RNA in the third trimester (at 38 weeks + 5 days and 38 weeks + 1 day of gestation), accompanied by typical dengue symptoms, including fever, headache, arthralgia, rash, or liver dysfunction. Neither the uninfected nor the DENV‑infected cases had any underlying diseases, and all neonates were asymptomatic at delivery. All human placental specimens were obtained after full‑term delivery. Upon admission, the patients provided written informed consent for the use of de‑identified discarded tissue for research purposes. This study used de‑identified residual tissue samples.

### Mice, cells, and viruses

*Ifnar1*^*-/-*^ mice (A6 mice) were bred in our laboratory. The mice were kept in a specific pathogen-free animal facility at Capital Medical University. Virgin female A6 mice,8–12 weeks of age, were mated with sexually mature fertile A6 male mice. The first day of gestation was determined by the observation of a vaginal plug [embryonic day (E) 0.5; term = E18.5]. At E12.5, pregnant A6 mice were challenged with 10^5^ plaque-forming units (PFU) DENV-2 (strain TR1751) through footpad injection and subsequently sacrificed on E15.5 and E18.5. For inhibition test of small molecule drugs, *Ifnar1*^*-/-*^ pregnant mice were injected with vehicle or SB225002 (5mg/kg, dissolved in DMSO) daily from E12.5 to E17.5 after infected with 10^5^pfu DENV-2. SB225002 (HY-16711) was purchased from MedChemExpress.

C6/36 cells, an *Aedes albopictus* mosquito cell line, used for DENV-2 propagation were maintained in RPMI 1640 medium with 10% fetal bovine serum (FBS) at 28°C. Vero cells were cultured in MEM medium supplemented with 5% FBS, which were used to determine the titer of DENV-2 by plaque formation assay.

### Hematoxylin and eosin staining

The fresh tissues were fixed in 4% paraformaldehyde and embedded in paraffin blocks through an automated tissue processor (Leica, Germany). 5µm slices were prepared according to the standard method. For morphological analysis, the slices were stained with Hematoxylin and Eosin (H.E.) and observed under microscopy. The level of placental red blood cell perfusion was assessed by comparing the proportion of placental red blood cells to the total placental area. The proportion of placental red blood cells was analyzed using ImageJ software.

### Immunohistochemistry (IHC) staining

For detecting immunocytes infiltration and placental microvessels, the sections were subjected to immunohistochemistry staining (IHC). The sections were incubated with anti-CD34(1:400, Abcam, Ab81289), anti-CXCL2 (1:200, Invitrogen, PA5–47015), anti-DENV NS1 (1:200, Abcam, Ab41616), anti-F4/80 (1:400, Abcam, Ab111101) or anti-Ly6G antibody (1:200, Abcam, Ab238132) at 4°C overnight. The sections were then stained with a secondary horseradish peroxidase (HRP)-conjugated goat anti-rabbit or mouse antibody (PV-9001, ZSGB-BIO) for 20 min at room temperature. The DAB (ZLI- 9018, ZSGB-BIO) was used to visualize the reaction as a chromogen. Finally, after counterstaining with hematoxylin, sections were observed under microscope.

### Fluorescence in situ hybridization (FISH)

Fluorescence in situ hybridization (FISH) was performed to detect DENV-2 RNA in paraffin-embedded sections. The samples included livers and placentas from DENV-2‑infected or uninfected pregnant mice. Liver sections from DENV-2‑infected pregnant mice served as positive controls, while sections from uninfected mice served as negative controls. A fluorescent probe targeting the DENV-2 Tr7151 strain (designed based on the viral genome sequence) was synthesized by Sangon Biotech (Shanghai, China) and labeled with 488 fluorescence at the 5′ end. All other reagents were obtained from a commercial FISH kit (Beyotime, China; catalog No. R0306S), and the procedure was carried out according to the manufacturer’s instructions.

### multi-Immunohistochemistry (mIHC) staining

To detect PF4+ macrophages and the expression of the chemokine CXCL2 within these cells in the placenta, multiplex immunohistochemical staining for PF4, F4/80, and CXCL2 was performed. The staining was carried out using a four-color multiplex immunofluorescence kit (AFIHC024, Hunan Aifang Biotechnology, China) based on Tyramide Signal Amplification (TSA) technology, according to the manufacturer’s instructions. Specifically, deparaffinized sections underwent antigen retrieval and were blocked for endogenous peroxidases, followed by blocking with 2% BSA. The sections were then incubated with primary antibodies at 4°C overnight. On the following day, secondary antibodies were applied, followed by TSA staining. Subsequently, the sections were subjected to antigen retrieval buffer for 30 minutes to strip off unbound antibodies. This cycle—starting with a new round of blocking, primary antibody incubation, secondary antibody incubation, and TSA staining was repeated until all three antibodies had been stained. Finally, coverslips were mounted on each slide using an antifade mounting medium containing DAPI (ZLI-9557, ZSGB-BIO, China), and the slides were examined under a confocal laser microscope. The primary antibodies used included: Rabbit anti-F4/80 antibody (1:200, Abcam, ab111101); Rabbit anti-PF4 antibody (1:400, Abcam, ab303494); Rabbit anti-CXCL2 antibody (1:400, Invitrogen, PA5-47015). All other reagents used were provided within the kit.

Human placental sections were stained using the same multiplex immunohistochemistry protocol as mentioned above in staining for mouse sections. For PF4⁺ macrophages and IL8, the primary antibodies were: rabbit anti-human PF4 (1:200, Invitrogen, MA5-38089), rabbit anti-human CD163 (1:200, Abcam, ab182422), and rabbit anti-human IL8 (1:200, Abcam, ab322732). For CXCR2⁺ neutrophils, the primary antibodies were: rabbit anti-human CXCR2 (1:200, Invitrogen, PA5-100951), rabbit anti-human CD66b (1:200, Invitrogen, PA5-104296), and rabbit anti-human PF4 (1:200, Invitrogen, MA5-38089). All other reagents were from the same kit as used for mouse staining.

### Sucrose density gradient centrifugation and Transwell migration assay

After euthanizing A6 mice, femurs were isolated and bone marrow cells were flushed out using a syringe. The cell suspension was then subjected to sucrose density gradient centrifugation, and cells localized at the neutrophil layer were collected.

A transwell migration assay was used to evaluate the role of the CXCL pathway in recruiting neutrophils to the placenta. Briefly, serum-starved neutrophils (1 × 105 per insert) were pre-incubated with or without 200 nM SB225002 (a specific CXCR2 inhibitor) for 2 hours, then seeded into the upper chamber of a Transwell upper insert containing a porous membrane. The lower chamber was filled with either uninfected or DENV-2-infected placental homogenate. After a 4-hour incubation, cells that had migrated to the lower surface of the membrane were fixed, stained, and counted under a microscope.

### Flow cytometry

To analyze the proportion of CXCL2^+^ neutrophils within the placenta, single-cell suspensions prepared from mouse placental tissue were first incubated with an anti-CD16/32 antibody (BD pharmingen, 553142) for Fc receptor blocking. Subsequently, the cells were stained with antibodies anti-mouse CD45 (AF700, 147715, BioLegend), CD11b (FITC, 101205, BioLegend), and Ly6G (APC, 127613, BioLegend). For intracellular CXCL2 staining, cells were fixed and permeabilized with the Cyto-Fast Fix/Perm Buffer Set (426803, BioLegend) for 20 min at 4°C, followed by incubation with PE-conjugated anti-mouse CXCL2 (orb485874, Biorbyt).

### Quantification of cytokines in mice placenta

According to the manufacturer’s specifications, the placental cytokines were analyzed using QAM-CHE-1 (CXCL2, CXCL1, CCL22, CCL9, CCL21b, CXCL16, CCL25, CX3CL1, CCL24, CCL12, CCL2, CCL11, CCL1, CXCL4, CXCL9, CXCL11, CXCL5, CCL27, CXCL13, CCL20, CCL19, CCL5, CXCL12, CCL17, CCL3) (RayBiotech, Norcross, GA, USA).

Fluorescence intensity was collected using an Axon Scanner 4000B with GenePix software. The detection limits for the cytokines are available on the manufacturer’s website.

### Single-cell RNA sequencing (scRNA-seq)

Placental tissues from uninfected or DENV-2-infected mice were placed in MACS C-Tubes (Miltenyi Biotec, Germany) containing MACS Tissue Storage Solution (Miltenyi Biotec, Germany) and subsequently delivered to LC-Biotechnology (Hangzhou, China) for single-cell RNA sequencing. Following tissue dissociation and erythrocyte lysis, single-cell suspensions devoid of red blood cells and cellular debris were prepared. Cell viability was assessed using trypan blue exclusion, and suspensions with >85% viability were processed for concentration adjustment (700–1200 cells/μL) using an automated cell counter. Single cells were captured via microfluidics-based technology on a Chromium Controller (10× Genomics, USA) following the manufacturer’s protocol. cDNA amplification and library preparation were subsequently performed, and libraries were sequenced on an Illumina NovaSeq 6000 platform. Raw sequencing data were demultiplexed, barcode-processed, and analyzed for 3′ gene expression quantification using Cell Ranger software. scRNA-seq reads were aligned to the Ensembl GRCm38 reference genome, followed by dimensionality reduction, clustering, and advanced computational analysis using Seurat (v3.1.1).

For single-cell RNA sequencing data analysis, the established computational pipeline detailed in our previous work was followed [50]. Briefly, gene expression values were calculated using the LogNormalize method via the NormalizeData function in Seurat. Principal Component Analysis (PCA) was subsequently performed on the scaled data. The optimal number of principal components for downstream analysis was determined based on the elbow plot of standard deviations. These selected components were utilized for two-dimensional projection of cells using Uniform Manifold Approximation and Projection (UMAP). To identify distinct cell populations, a graph-based clustering approach was employed through the construction of a weighted Shared Nearest Neighbor (SNN) graph. Marker genes for each resultant cluster were then identified with the Bimod likelihood-ratio test using the FindAllMarkers function. Biological functions were interrogated through Kyoto Encyclopedia of Genes and Genomes (KEGG) and Gene Ontology (GO) enrichment analyses of the differentially expressed genes, which were conducted using the clusterProfiler R package (v4.2.2). Finally, to infer intercellular communication networks, the CellChat tool (v1.6.1) was employed, whereby communication probabilities were calculated based on a curated ligand-receptor interaction database and significant interactions were identified through permutation testing.

### Single nucleotide polymorphism sequencing (SNP-seq)

To determine the cellular origin of placental cells, single-nucleotide polymorphism (SNP) profiling was performed alongside single-cell RNA sequencing of the placenta. This allowed for the recording of SNP information for each individual cell. Concurrently, SNP sequencing was conducted on the maternal mice and the livers of the corresponding fetuses to establish the SNP profiles of both the mothers and the fetuses. The cellular origin of placental cells was then determined based on the unique SNP markers that distinguished the maternal and fetal genomes. The analysis was performed using the Demuxlet software, the algorithm of which has been previously published (see reference).

### Quantification and statistical analysis

GraphPad Prism 8.0 was used for data visualization and statistical analyses. The normality was analyzed by a Kolmogorov-Smirnov test. The data with normal distributions were analyzed by t test or one-way analysis of variance (ANOVA) followed with post hoc tests, while those with abnormal distributions or heterogeneity of variance were analyzed by the nonparametric Mann-Whitney test or Kruskal-Wallis one-way ANOVA. The data are presented as the means ± standard deviation or means ± standard error of the mean (SEM). *P < 0.05; **P < 0.01 were considered statistic difference, significant statistical difference and highly significant statistical difference, respectively.

## Supporting information

S1 Fig Single cell sequencing reveals DENV-2 infection in placenta. (A) Dot plot visualizing the percentage of DENV-2 RNA positive cells (dot size) across all annotated cell populations in E18.5 placentas. (B) A screenshot from the Integrative Genomics Viewershowing the alignment of sequencing reads to the DENV-2 reference genome. (C) Detection of DENV-2 NS1 antigen (red, 594 nm) and viral RNA (green, 488 nm) by immunofluorescence and FISH, respectively, in paraffin-embedded liver and placenta sections from uninfected and DENV-2-infected pregnant mice. Nuclei were stained with DAPI (blue). Scale Bar = 50 µm arrow. white arrow indicates DENV-2 NS1 and RNA positive cell.
(TIF)

S2 Fig The accumulation of CXCL2 + neutrophils in the placenta after DENV-2 infection. (A) Bar plot quantifying the contribution of monocyte/macrophage and neutrophil clusters to CXCL signaling. (B) Representative immunohistochemistry images showing CXCL2 expression in placental sections. Black arrows indicate CXCL2-positive cells with segmented nuclei. (C) Flow cytometry plots gated on CD11B^+^Ly6G^+^ cells, showing representative profiles of CXCL2^+^ cells. (D) Statistical analysis of CXCL2^+^ neutrophils in placentas. (E) Representative images of migrating neutrophils on the transwell membrane and quantification of their numbers (n = 3 independent experiments). Scale bar: 50 µm. In panel D and E, data was presented as the mean and Students’ t-test was used for statistical analysis. *, p < 0.05; **, p < 0.01; ns, not significant.
(TIF)

S3 Fig Single‑cell sequencing reveals that neutrophil infiltration is specific pathological features of DENV‑2‑infected placenta compared to ZIKV infection. UMAP visualization of single-cell transcriptome sequencing data from ZIKV-infected placenta, DENV-2-infected placenta, and their respective control placenta samples. Cells are color-coded by cell type annotations as indicated. A red square highlights the Granulocyte population.
(TIF)
